# Adenomyosis in infertile women: prevalence and the role of 3D ultrasound as a marker of severity of the disease

**DOI:** 10.1186/s12958-016-0185-6

**Published:** 2016-09-20

**Authors:** J. M. Puente, A. Fabris, J. Patel, A. Patel, M. Cerrillo, A. Requena, J. A. Garcia-Velasco

**Affiliations:** 1 Department of Reproductive Medicine, IVI Madrid, Av del Talgo 68, 288023 Madrid, Spain; 2 Department of Reproductive Medicine, IVI Madrid, Rey Juan Carlos University, Madrid, Spain

**Keywords:** Adenomyosis, Infertility, Ultrasound diagnosis, Three-dimensional ultrasound

## Abstract

**Background:**

Adenomyosis is linked to infertility, but the mechanisms behind this relationship are not clearly established. Similarly, the impact of adenomyosis on ART outcome is not fully understood. Our main objective was to use ultrasound imaging to investigate adenomyosis prevalence and severity in a population of infertile women, as well as specifically among women experiencing recurrent miscarriages (RM) or repeated implantation failure (RIF) in ART.

**Methods:**

Cross-sectional study conducted in 1015 patients undergoing ART from January 2009 to December 2013 and referred for 3D ultrasound to complete study prior to initiating an ART cycle, or after ≥3 IVF failures or ≥2 miscarriages at diagnostic imaging unit at university-affiliated private IVF unit. Adenomyosis was diagnosed in presence of globular uterine configuration, myometrial anterior-posterior asymmetry, heterogeneous myometrial echotexture, poor definition of the endometrial-myometrial interface (junction zone) or subendometrial cysts. Shape of endometrial cavity was classified in three categories: 1.-normal (triangular morphology); 2.- moderate distortion of the triangular aspect and 3.- “pseudo T-shaped” morphology.

**Results:**

The prevalence of adenomyosis was 24.4 % (*n* = 248) [29.7 % (94/316) in women aged ≥40 y.o and 22 % (154/699) in women aged <40 y.o., *p* = 0.003)]. Its prevalence was higher in those cases of recurrent pregnancy loss [38.2 % (26/68) vs 22.3 % (172/769), *p* < 0.005] and previous ART failure [34.7 % (107/308) vs 24.4 % (248/1015), *p* < 0.0001]. The presence of adenomyosis has been shown to be associated to endometriosis [35.1 % (34/97)]. Adenomyosis was diagnosed as a primary finding “de novo” in 80.6 % (*n* = 200) of the infertile patients. The impact on the uterine cavity was mild, moderate and severe in 63.7, 22.6 and 10.1 % of the cases, respectively.

**Conclusions:**

Our results indicate that adenomyosis is a clinical condition with a high prevalence that may affect the reproductive results. The described severity criteria may help future validating studies for better counseling of infertile couples.

## Background

The term adenomyosis was first used in 1972 [1] et al. to describe the presence of both endometrial glands and stroma deep within the myometrium. This condition is associated with hypertrophy and hyperplasia of the subjacent muscle cells [2], which may ultimately result in an altered size and globulous morphology of the uterus, although the clinical signs and symptoms are variable. There is presently a lack of precise data regarding adenomyosis prevalence among general gynecologic patients [3], as well as about the impact of this condition within the reproductive context.

Adenomyosis is linked to infertility, but the mechanisms behind this relationship are not clearly established [4]. Similarly, the impact of adenomyosis on ART outcome is not fully understood, as data are scarce and there are contradictions within the available evidence. While some groups report that adenomyosis negatively impacts outcomes of IVF [5–8], others have not found this negative association [9–12]. Adenomyosis has been associated with a higher prevalence of miscarriage [11] and with a generally worse perinatal outcome [13]. There is a well-established association between endometriosis and adenomyosis, such that adenomyosis is a plausible contributing factor to infertility among endometriosis patients [14]. Also, women are more commonly diagnosed with adenomyosis during the later stages of reproductive age [15, 16]. Thus, we might expect an age-related increase in adenomyosis prevalence based on the trend of postponing maternity in the western world.

The majority of published reports describing adenomyosis prevalence rely on pathologic analysis of surgical specimens [17], which is not an option in infertile patients. Compared to pathology, modern imaging methods using transvaginal ultrasound and, even better, MRI with T2-weighted images enable a more detailed evaluation of the changes in the smooth muscle cells [18]. Thus, imaging is an excellent tool for patient evaluation and management.

In the present study, our main objective was to use ultrasound imaging to investigate adenomyosis prevalence and severity in a population of infertile women, as well as specifically among women experiencing recurrent miscarriages (RM) or repeated implantation failure (RIF) in ART.

## Methods

We performed a transversal study that included 1015 patients attending the Diagnostic Imaging Unit at our institution between January 2009 and December 2013. Patients were referred to this unit prior to initiating an ART cycle. The immense majority of the referred patients were those who showed some pelvic abnormalities in a conventional 2D ultrasound, and patients with recurrent miscarriage of repeated failure of ART. Table 1 summarizes the reasons for referral, although patients commonly had more than one indication. Table 2 summarizes general population data, such as age, smoking habit, BMI, and previous pregnancies.

**Table 1:** Main indication for referral to the Diagnostic Imaging Unit

	N	Percent
ART failure	305	30.0
Recurrent miscarriage	68	6.7
Endometriosis	28	2.8
Ovarian cyst	62	6.1
Tubal abnormalities	116	11.4
Suspected fibroid	156	15.4
Mullerian malformation	55	5.4
Suspected endometrial polyp	31	3.1
Unexplained infertility	115	11.3
Others	79	7.8
Total	1015	100.0

**Table 2:** Epidemiological data from the study population

Variable	Adenomyosis n (%)	OR	*P*
Age < 40 y	154/699 (22.0)	0,67 (0,50–0,91)	*p* < 0.01
Age ≥ 40 y	94/316 (29.7)	1,50 (1,11–2,02)	*p* < 0.01
Smokers %	25/93 (26.8)	1,1 (0,70–1,84)	*p* = 0.56
Pregnancies			
0	233/963 (24.1)	0,7 (0,38–1,31)	*p* = 0.44
≥ 1	13/46 (28.2)	1,23 (0,63–2,38)	*p* = 0.53
Recurrent miscarriage (RM)	26/68 (38.2)	2,03 (1,21–3,39)	*p* < 0.005
ART failure	107/305 (34.7)	2,14 (1,59–2,89)	*p* < 0.0001
Endometriosis	34/97 (35.1)	1,77 (1,14–2,77)	*p* = 0.01
Fibroids	48/266 (18)	0,60 (0,42–0,85)	*p* < 0.005

A patient was considered to have undergone recurrent miscarriage if she had at least two consecutive intrauterine pregnancy losses confirmed by ultrasound or pathology [19]. Repeated implantation failure was defined as the failure of two good quality double-embryo transfers, independent of maternal age. This study was approved by the institutional IRB (1407-MAD-052-HM).

**Fig. 1:**
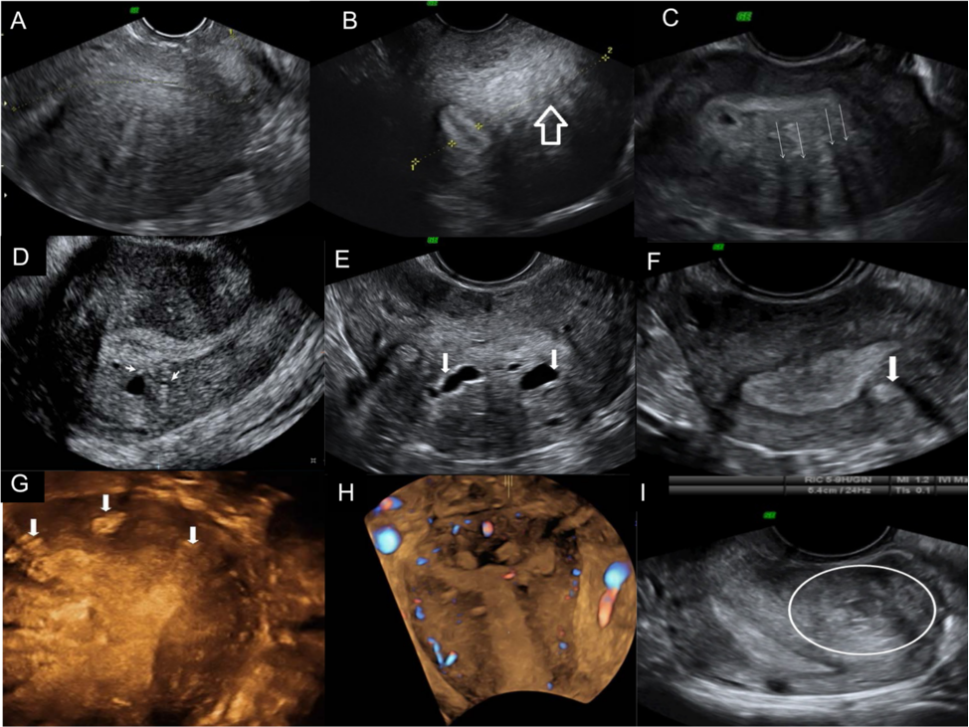
Ultrasonographic diagnostic criteria for adnomyosis. **a** Globulous aspect of the uterus. **b** Uterine asymmetry. Longitudinal section of a retroverted uterus, where the posterior uterine wall is clearly thicker than the anterior wall. **c** Heterogeneous myometrial texture. Transversal section of the uterus at the fundus level, where hypoechoic areas with radial pattern can be seen (*arrows*). **d** Linear striations. In this sagital section of an anteverted uterus thin hyperecogenic lines cross the myometrial thickness, visible from the endometrial-myometrial interphase. **e** Intramyometrial cysts. Transversal section of the uterus at the fundus level with sonoluscent images distributed in posterior wall of the myometrium. **f** and **g**, **h** Hyperechogenic nodules. Transversal (**f**) and coronal (**g**, **h**) sections of the uterus at the fundus level where hyperechogenic Intramyometrial areas can be observed (*arrows*). **i** Adenomyoma. Longitudinal section of a retroverted uterus with heterogeneous nodular mass lacking well-defined margins in the posterior wall

Our adenomyosis diagnostic criteria were based on previously published criteria (Fig. 1) [20–28] that have been used in other recent studies [3]. Adenomyosis was diagnosed in patients showing the presence of one or more of the following criteria: 1) globulous aspect of the uterus, defined as a global increase in uterine myometrial thickness not caused by fibroids or other pathologic uterine condition, 2) uterine asymmetry, defined as thickening of the anterior uterine wall vs. the posterior, or vice versa, 3) heterogeneous myometrial texture, or alternating hyperechogenic and hypoechogenic areas in terms of myometrial thickness without a precise margin, along with thin acoustic shadows with a radial pattern that are not induced by fibroids or intramyometrial hyperechogenic foci, 4) irregular endometrium–myometrium interphase, or lack of a clearly visualized neat contour of the endometrial basal layer and the underlying myometrium, with no or incomplete visualization of the junction zone (JZ), 5) presence of intramyometrial cysts, or anechoic areas with myometrial thickness of ≥1 mm and negative for color Doppler (power Doppler or high-definition Doppler), 6) linear striations from the endometrium to the myometrium, or hyperechogenic lines crossing the myometrial thickness, visible from the endometrial–myometrial interphase, and/or 7) adenomyoma, defined as a heterogeneous nodular mass lacking well-defined margins and without internal calcifications.

Clinical data was obtained from the electronic medical records stored in our database, as well as prospectively with the stored 3D volumes. Transvaginal ultrasounds were performed, complemented with abdominal ultrasound when required. All ultrasounds were performed by the same experienced explorer (JMP) to reduce the inter-observer variability associated with adenomyosis diagnosis by transvaginal ultrasound. All scans were performed between days 8 and 28 of the menstrual cycle to evaluate endometrial thickness. Both 2D and 3D scans were performed in all cases, following the manufacturer’s specific recommendations (Voluson 730 Expert, GE Healthcare, Milwaukee, WI, USA) using a 2.9- to 10-MHz transvaginal probe.

**Fig. 2:**
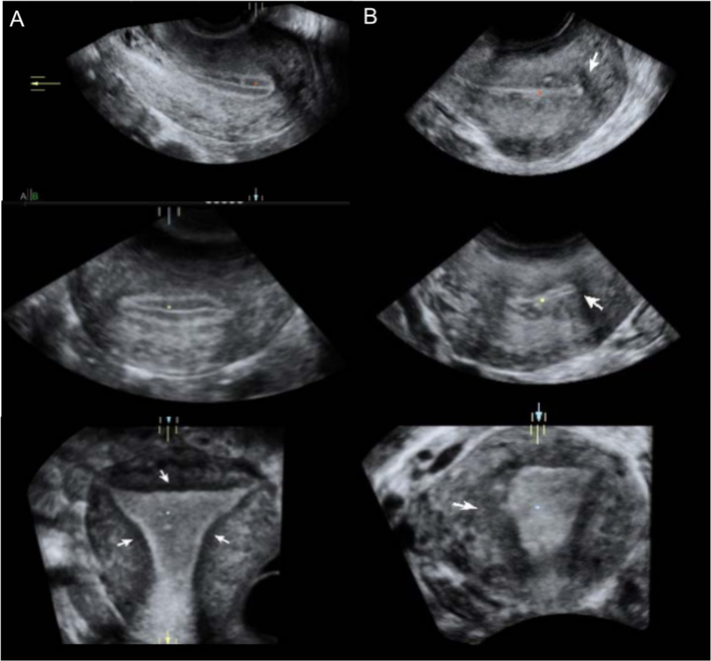
Evaluation of the junction zone (JZ). Multiplanar view in volume contrast image (VCI) mode attaining images with 2 mm slice thickness. Sagital, transversal and coronal views of a retroverted uterus **a** Normal JZ, observed as hypoechogenic area surrounding all endometrial thickness (*arrows*). **b** Thickened, irregular JZ

**Fig. 3:**
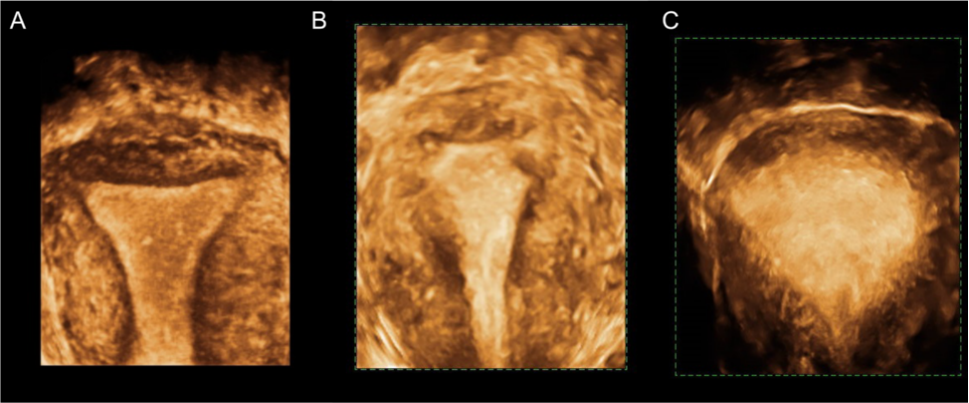
Evaluation of the JZ using 3D surface reconstruction mode. **a** Normal JZ. **b** and **c** thickenned, irregular JZ, where it is not possible to adequatly differentiate the endometrial-myometrial transition

We evaluated the JZ using 3D ultrasound with a multiplanar view in volume contrast imaging (VCI) mode [29], attaining images of the coronal and sagittal planes with a 2-mm slice thickness (Fig. 2). We also used surface reconstruction mode (Fig. 3). A 90° angle was formed between the ultrasound beam and the axis of the endometrial cavity. Ultrasonographic examination started with bi-dimensional evaluation of the uterus in the sagittal section, and then in the transversal section. In this section, the myometrium was also examined and images/videos were stored. Power Doppler HD was used to evaluate the endometrial/myometrial mapping, and we obtained the pulsatility index (PI) of both uterine arteries [30]. We continued the examination with a coronal section, and uterine volume was obtained from the sagittal section, including the entire uterus and storing at least one 3D volume. If volume acquisition from a sagittal plane was suboptimal, the volume was instead obtained from a transversal section.

**Fig. 4:**
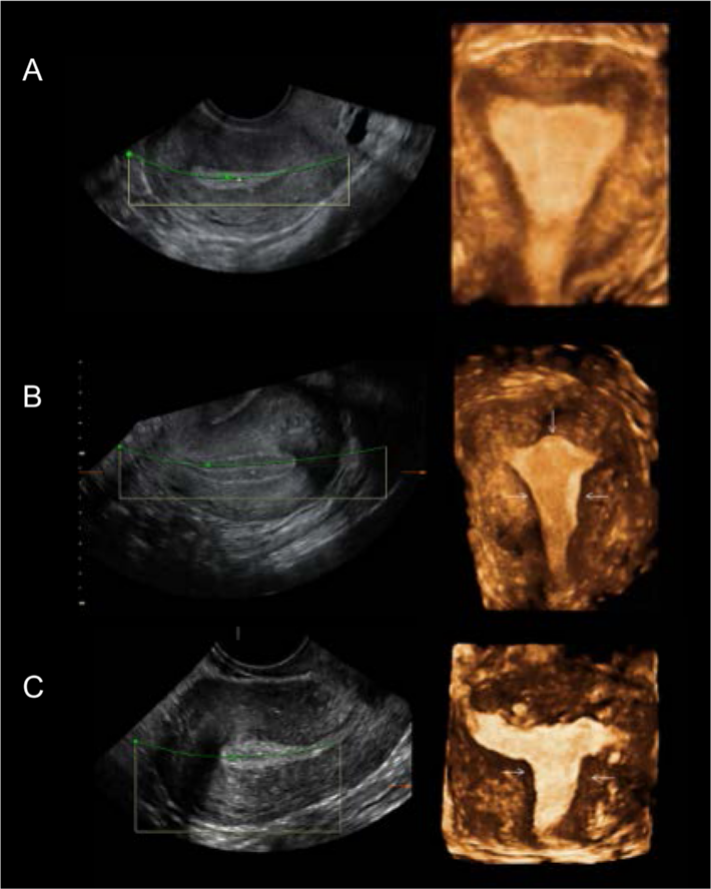
Evaluation of the uterine cavity using 3D reconstrution mode in women diagnosed with adenomyosis. **a** Normal morphology of the uterine cavity, where JZ is thickenned and irregular, but the uterine cavity maintains its triangular shape. **b** Moderate alteration of the uterine cavity, with a convex shape in the upper cavity, and a narrowing of the lateral walls (*arrows*); myometrium is hypertrophic and irregular. **c** Severe modification of the uterine cavity, with funneling of the lateral walls (*arrows*), adopting a T-shaped morphology (*arrows*). Multiple hypoechogenic areas can be observed within the endometrium

As summarized in Fig. 4, the impact of adenomyosis-induced endometrial cavity damage was classified in three categories: 1.- normal cavity, when the cavity retains its triangular morphology, 2.- moderate distortion of the triangular aspect of the endometrial cavity without reaching a “pseudo T-shaped” morphology, and 3.- “pseudo T-shaped” morphology. Endometrioma was diagnosed using IOTA criteria, based on the observation of a well-defined cystic structure with a thick capsule and low-intensity echoes (ground glass), and with a homogenous aspect of the interior [31, 32]. To diagnose deep endometriosis, we used a combination of clinical symptoms (e.g., pain during ultrasonographic evaluation) and sonographic findings (e.g., stellate hypoechoic or isoechogenic solid masses with irregular outer margins that are power Doppler-negative in the anterior/posterior compartment) [33–36].

Statistical analysis was performed using SPSS (SPSS Inc., Chicago, IL, USA). Data were expressed as absolute values and percentages. Qualitative variables were analyzed using the chi-square test, calculating the odds ratio (OR) and confidence interval (CI). Significance was set at 95 %. Continuous variables were expressed as mean and standard deviation, and were analyzed by Student’s t-test. A *P* value of <0.05 was considered significant.

## Results

Within our population of women referred to the Diagnostic Imaging Unit, the adenomyosis prevalence was 24.4 % (*n* = 248/1015). Among all 248 women diagnosed with adenomyosis, 48 (19.4 %) had been previously diagnosed with adenomyosis, whereas 200 were new cases diagnosed in our unit. Women diagnosed with adenomyosis had a higher mean age (38.3 ± 4.1 years) than women without adenomyosis (37.2 ± 4.7 years), but this difference was not significant (*P* = 0.99). Adenomyosis prevalence was significantly higher among women ≥ 40 years of age (29.7 %, *n* = 94/316) compared to among women < 40 years of age (22 %, *n* = 154/699) (*P* = 0.003). Mean BMI was significantly lower among women with adenomyosis (20.9 ± 4.5) than among women without adenomyosis (21.8 ± 3) (*P* = 0.003).

Smoking habits did not significantly differ between groups, with smoking reported by 25 of the 248 women with adenomyosis (10.1 %) compared to 68 of the 767 women without adenomyosis (8.9 %) (*P* = 0.56). We also found no between-group differences in parity status, as 94 % of women with adenomyosis were nulliparous compared to 95.2 % of women without adenomyosis (*P* = 0.44).

Among the study participants, 68 women were referred to our unit with RM as their main indication, and this subgroup of patients showed a higher prevalence of adenomyosis (38.2 % [26/68] vs. 22.3 % [172/769], *P* < 0.005). A total of 308 participants showed RIF, and their adenomyosis prevalence was 34.7 % (107/308), which was significantly higher compared to the general prevalence (24.4 %, 248/1015) (*P* < 0.0001).

Among the 97 patients diagnosed with endometriosis, 35.1 % (34/97) were also diagnosed with adenomyosis. Fibroids were diagnosed in 266 patients, of whom 48 (18.0 %) also had adenomyosis (Table 2). Regarding the impact of adenomyosis on uterine morphology, of the 248 cases of adenomyosis, 167 (63.7 %) showed mild uterine damage, 56 (22.6 %) showed moderate morphological damage, and 25 (10.1 %) showed severe damage, i.e., a “pseudo T-shaped” uterine cavity (Table 3).

**Table 3:** Impact of adenomyosis on cavity distortion

Cavity distortion	N	Percent
No impact/mild	167	67.3
Moderate	56	22.6
Severe	25	10.1
Total	248	100.0

## Discussion

Adenomyosis diagnosis through imaging techniques remains strongly operator dependent, and is much more frequent among women already known to be suffering from specific conditions, such as infertility, menorrhagia, and/or dysmenorrhea. Our present results showed that infertile patients had a high prevalence of newly diagnosed adenomyosis. Furthermore, adenomyosis was strongly related to maternal age and, as demonstrated, may compromise reproductive outcome.

Adenomyosis can be diagnosed both by transvaginal ultrasound [27, 37] and MRI [38]. In recent years, the diagnostic accuracy of ultrasound for adenomyosis has improved substantially, mainly due to improvements of technology and higher awareness of the ultrasonographers. With the addition of 3D ultrasound and a closer evaluation of the transition zone from the endometrium to the myometrium (the JZ), ultrasound evaluation for adenomyosis is reproducible and may show improved diagnostic accuracy [3, 29, 39].

MRI is considered the gold standard for adenomyosis diagnosis. However, transvaginal ultrasound shows a good correlation and strong agreement with MRI [40]. Ultrasound has the advantages of lower cost and easier access compared to MRI. Additionally, transvaginal ultrasound allows the operator to obtain clinical data from the patient (i.e., regarding pain during the examination) or images suggestive of pelvic adhesions [33, 41]. There are some studies comparing MRI versus ultrasound [21, 42–44]. Champaneria et al. [45], performed a systematic review and they found that both MRI and ultrasound had good diagnostic accuracy but MRI performed better than ultrasound. Thus, transvaginal ultrasound is an ideal screening test [46], with MRI reserved as a back-up technique to be used in cases with unclear diagnosis [42] or when multiple/large fibroids complicate the sonographic examination [43].

It is likely that the diagnostic specificity of ultrasound is better in severe cases and in cases with other concomitant conditions, such as endometriosis [47], when compared with less severe cases. Therefore, it is strongly recommended to establish severity criteria, as suggested by Vercellini et al. [46], and to perform trials to compare diagnostic specificity and sensibility according to adenomyosis severity. This could potentially enable earlier identification of cases with a poor reproductive prognosis. Although there is presently no evidence suggesting the potential benefit of medical or surgical intervention in terms of fertility prognosis, establishing severity criteria could help clinicians to better counsel their patients regarding their chances of achieving a live birth.

Investigating the adenomyosis prevalence within the context of assisted reproduction is difficult, as it is often impossible to correlate the imaging diagnosis with the pathologic report, as can be done in other areas of gynecology. This may partially explain the huge disparity among prevalence reported in the literature—which range from 16 to 66 % depending on the type of patients included, the diagnostic criteria, and/or the number of sections evaluated [48]. Our present study showed a high global adenomyosis prevalence of 24 %, which compares favorably with the prevalence found among symptomatic women attending a gynecologic clinic [3]. To the best of our knowledge, our present study is the largest adenomyosis screening among infertile women.

Strikingly, among all of the adenomyosis diagnoses in this study, 4 out of 5 patients had not been previously diagnosed. This is surprising, particularly considering that these women had undergone multiple previous transvaginal scans by the time they reached the Imaging Unit. Ultrasonographers should have a higher awareness of the relevance of adenomyosis among gynecologic patients, especially those who are being examined for infertility. Given the strong association between adenomyosis and infertility, we agree with other groups [49] that adenomyosis should be part of the differential diagnosis in the first consultation of an infertile patient.

Our results showed a significantly higher prevalence of adenomyosis in women over 40 years of age, as has been previously described [50, 51]. This suggests that adenomyosis could potentially be linked to uterine senescence. However, a subgroup of young women shows adenomyosis that is frequently associated with endometriosis. Among patients with adenomyosis in our population, 35 % showed concomitant adnexal or deep endometriosis. This relationship should be carefully investigated, as adenomyosis may contribute differently to infertility in this particular patient subgroup, potentially explaining the large number of young women found in our series.

We detected no relationship between smoking habit and adenomyosis, and the previously reported link between tobacco and endometriosis is controversial [50]. Although multiparity has been described as a risk factor for adenomyosis [3, 50], we found no such association. However, the large proportion of nulliparous patients was expected, as our study population comprised infertile women. In our series, being diagnosed with adenomyosis was not an additional risk factor for having uterine fibroids. These conditions coexisted in only 18 % of our patients, similar to findings described in the literature [3]. It should be noted that large or multiple fibroids may confound the diagnosis of adenomyosis, such that is has been suggested that MRI should be used in these patients to improve diagnostic accuracy [43].

We found a significantly higher adenomyosis prevalence among our patients with recurrent miscarriage. Having had at least two miscarriages was associated with being diagnosed with adenomyosis. The patients referred to the Imaging Unit specifically due to RM had a high adenomyosis prevalence (38.2 %). This may have been because these patients had undergone larger numbers of interventions that may have damaged the endometrium–myometrium interphase, which facilitates glandular epithelial endometrium migration [51]. It is also possible that women with adenomyosis may have a higher risk of miscarriage due to a uterine factor [11]. We may speculate, as others [52] that adenomyosis, due to abnormal trophoblast invasion of the spiral and radial arteries, could lead to defective placentation that facilitates preterm delivery, small-for-gestational-age fetuses, and puerperal hemorrhage.

Similarly, we found a higher adenomyosis prevalence among women with RIF, which may suggest poorer endometrial receptivity among patients with adenomyosis. There is contradictory evidence regarding this matter—with some authors describing poorer pregnancy rates after ART among women with adenomyosis [5–8, 53], and others not finding any such association [9–12]. These discordant results may be partially explained by the limited sample sizes in most of the previous underpowered studies, as well as the varying assisted reproductive techniques utilized. The donor egg model would be optimal for such studies, as it minimizes the impact of embryo quality while emphasizing the influence of adenomyosis. Furthermore, previous results have not been analyzed according to the disease severity, which is likely an important factor.

Here we propose easily reproducible screening criteria of severity by which uterus morphology is classified into three categories based on 3D transvaginal ultrasound results. This system allows the evaluation of pregnancy rates according to disease severity. Smooth muscle cell hyperplasia of the JZ, or “myosis,” is not always associated with glandular invasion [18, 20]. Thus, uterine evaluation should always incorporate coronal sections, which allow examination of both JZ thickness and funneling of the uterine cavity. To the best of our knowledge, there is no published evidence that this progression has been described. This is just based on personal observation and on the fact the DES was not used in our country at the time it was used in other parts of the world, so the relationship with DES exposure –although possible- is highly unlikely. Adenomyosis-associated modification of the uterine cavity could have a negative impact on natural fertility.

Strengths of our study include the homogeneous infertile patient population studied, which is the largest series investigated to date, and the fact that a single operator performed all scans, thus minimizing interobserver variation. Additionally, the systematic storage of 3D volumes allowed case reevaluation in instances of diagnostic uncertainty. The main limitation of the present study was that the Imaging Unit does not evaluate all infertile couples being treated at our institution—only those in whom pelvic pathology is suspected (RIF, RM, unclear 2D uterine morphology, etc.). Thus, the present results may overestimate the adenomyosis prevalence in the general infertile patient population. Additionally, the lack of pathologic confirmation after surgery may limit the accuracy of the diagnosis; however, this limitation shared with any other study performed in infertile women.

Adenomyosis treatment includes both medical and surgical management [54]. It should be noted that the choice of treatment is influenced by factors such as associated symptoms (dysmenorrhea, chronic pelvic pain or excessive bleeding) or coexistence with other benign diseases of the uterus such as endometriosis or fibroids. Given the scarce evidence available in the medical treatment of adenomyosis in the context of infertility, it appears that the use of GnRH analogues for 3–6 months could reduce both uterine size as well as endometriotic implants [55]. The surgical approach is exceptional in infertile patients since the excision of the adenomyotic nodules by different surgical techniques could weaken the myometrial wall, which is associated with a higher risk of uterine rupture during pregnancy.

## Conclusions

Our present results showed an elevated prevalence (24.4 %) of adenomyosis among infertile women. The advanced maternal age and the higher prevalence of endometriosis observed in infertile women most likely contributed to this higher adenomyosis prevalence. We further observed even higher adenomyosis prevalence in subsets of women with RIF and RM, supporting the possibility that adenomyosis may have a deleterious impact in reproduction. The described severity criteria may help future validating studies for better counseling of infertile couples.

## Abbreviations

ART: Assisted Reproductive Technology
IVF: In vitro fertilization
MRI: Magnetic resonance imaging
PI: Pulsatility index
RIF: Repeated implantation failure (RIF)
RM: Recurrent miscarriage
